# Early incidence of occupational asthma among young bakers, pastry-makers and hairdressers: design of a retrospective cohort study

**DOI:** 10.1186/1471-2458-10-206

**Published:** 2010-04-26

**Authors:** Thomas Rémen, Vincent Coevoet, Dovi-Stéphanie Acouetey, Jean-Louis Guéant, Rosa-Maria Guéant-Rodriguez, Christophe Paris, Denis Zmirou-Navier

**Affiliations:** 1 Institut National de la santé et de la Recherche Médicale U 954, School of Medicine, Nancy, France; 2 University Medical School, Nancy, France; 3 EHESP School of Public Health, Rennes, France

## Abstract

**Background:**

Occupational exposures are thought to be responsible for 10-15% of new-onset asthma cases in adults, with disparities across sectors. Because most of the data are derived from registries and cross-sectional studies, little is known about incidence of occupational asthma (OA) during the first years after inception of exposure. This paper describes the design of a study that focuses on this early asthma onset period among young workers in the bakery, pastry making and hairdressing sectors in order to assess early incidence of OA in these "at risk" occupations according to exposure duration, and to identify risk factors of OA incidence.

**Methods/Design:**

The study population is composed of subjects who graduated between 2001 and 2006 in these sectors where they experience exposure to organic or inorganic allergenic or irritant compounds (with an objective of 150 subjects by year) and 250 young workers with no specific occupational exposure. A phone interview focusing on respiratory and 'Ear-Nose-Throat' (ENT) work-related symptoms screen subjects considered as "possibly OA cases". Subjects are invited to participate in a medical visit to complete clinical and lung function investigations, including fractional exhaled nitric oxide (FE_NO_) and carbon monoxide (CO) measurements, and to collect blood samples for IgE (Immunoglobulin E) measurements (total IgE and IgE for work-related and common allergens). Markers of oxidative stress and genetic polymorphisms exploration are also assessed. A random sample of 200 "non-cases" (controls) is also visited, following a nested case-control design.

**Discussion:**

This study may allow to describ a latent period between inception of exposure and the rise of the prevalence of asthma symptoms, an information that would be useful for the prevention of OA. Such a time frame would be suited for conducting screening campaigns of this emergent asthma at a stage when occupational hygiene measures and adapted therapeutic interventions might be effective.

**Trial registration:**

Clinical trial registration number is NCT01096537.

## Background

The association between asthma and occupation is recognized since Antiquity [1] but our understanding of the pathophysiological mechanisms that are involved is still scanty. Definition of occupational asthma (OA) has been evolved along time. Recently, Bernstein [2] has described OA as "*a disease characterized by variable airflow limitation and/or airway hyperresponsiveness due to causes and conditions attributable to a particular occupational environment and not to stimuli encountered outside the workplace *[...]". This definition excludes "aggravated asthma", a pre-existent asthma which is exacerbated by working conditions.

Two forms of OA have been described: (i) the Reactive Airways Dysfunction Syndrome (RADS), which may be induced by acute inhalation of irritant substances; and (ii) the immunological type whose latency period after exposure is variable. RADS (which represents 5.5% to 9.5% of OA cases [3,4]) is a non-immunologic form of OA described by Brooks [5] as a persistent asthma syndrome following exposure to high levels of irritants, typically as a result of an accident occurring in the workplace or of a situation with poor ventilation and limited air exchange. The most frequent form of OA is the immunological form and arises after a latency period during which sensitization is acquired. The case of exposure to high molecular weight (HMW) agents (proteins >5000 daltons) differs from that implicating low molecular weight (LMW) agents (chemical products <5000 daltons), on account of their characteristics and the mechanisms that will eventually end up in OA.

Among HMW agents associated with OA, one can find several families of agents as, for example, animal-derived antigens, flour or pollen [6]. OA caused by exposure to HMW agents arises from mechanisms bounded to the production of IgE specific antibodies. This form of OA does not differ from non-occupational asthma associated with common pneumallergens in the environment [7]. In this category, bakers' asthma represents the first cause of OA in France [8] and one of the most current aetiology in countries of Western Europe [9-11]. Despite improvements in the implementation of occupational prevention measures, it affects an important proportion of workers in the bakery or pastry sectors. During the last twenty years, several authors have studied OA incidence among bakers with different methodologies, yielding incidence rates ranging from 0.334 to 2.46 cases per 1.000 person-years [3,9-17]. Three categories of agents are implicated: (i) flour (wheat, rye...) which is the main cause of baker's allergy, (ii) flour contaminants (storage mite, moulds,...) and (iii) substances added during bread making process (α-amylase, baker's yeast, soy lecithin,...). Their relative importance depends on flour characteristics, stocking conditions and exposure intensity [18].

Diisocyanates and their arrangement, chemical products or products used in health field are some examples of the different families of LMW agents known to date as possibly implicated in OA [6]. As opposed to HMW agents, the mechanisms involved are still discussed. Some LMW substances are able to induce OA through an IgE-dependant mechanism in a small proportion of persons. Among these substances are anhydrids (trimellitic and phtalic), diisocyanates, nickel and plicatic acid [18]. For others LMW substances, the physiopathology is more complex. Several characteristics of the condition suggest an immuno-allergic mechanism: the latency period between inception of exposure and occurrence of symptoms, or individual susceptibility. However, among affected subjects, IgE specific antibodies have not brought light for this category of LMW substances [19]. Hairdressing is one of the occupational sectors that is the most concerned by OA implicating exposure to LMW agents. In France, hairdressers represent the third category of workers with the highest risk of occupational asthma, following bakers/pastry makers and car painters, and the first category among females [3]. However, data about OA incidence are few for hairdressers [3,20]. In a retrospective Swedish nationwide study, Albin finds a higher asthma incidence during active years as a hairdresser (3.9 per 1.000 person-years) compared with the referents - women randomly selected from the general population (3.1 per 1.000 person-years; incidence rate ratio (IRR) = 1.2 [95% confidence interval 1.0-1.6]) [21]. While the incidence among hairdressers is smaller than in the bakery sector, several authors, as Ameille, observed an increasing trend: since the ONAP (Observatoire National des Asthmes Professionnels) was created in France in 1996, the proportion of OA in the hairdressing sector progressed from 5.5% in 1996 to 8% in 1998 and 10% in 2000 [22], calling for a better surveillance in this occupation.

Whether among bakers or hairdressers, incidence figures are inclined to be underestimated. Studies based on workers at work are exposed to the "healthy worker effect", a selection bias. Also, owing to the fact that administrative procedures are complex or that some workers do not prefer to engage administrative procedures by fear of losing their job, official statistics are very imperfect. Moreover, they tend to weaken the OA frequency because they do not consider subclinical or mild forms of asthma. Finally, longitudinal studies are rare (especially concerning LMW agents) and the effect of exposure seniority has not been assessed. Nevertheless, a few cohort studies have been conducted in a variety of occupations and tend to demonstrate that "*the most important risk for developing OA is the level and duration of exposure to agents capable of causing OA*" [23]. Several studies demonstrated the existence of a dose-response relationship between exposure intensity and the prevalence of immunological sensitization to various occupational agents [13,24-28].

Now, while occupational exposure plays the key role, other factors, related to personal or more general characters, contribute also to the onset of the disease, among which are nutrients intake and genetic characteristics. From a genetic point of view, associations were described between OA and certain antigens of class 2 implicated in the presentation of antigens in the immunizing cells [29-34] or some genotypes of the glutathione S-transferase and of the N-acetyltransferase [35-37]. In a recent review, genetic factors that predispose asthma have been broken down into three broad categories: immune and inflammatory (12 genes), atopic (3 genes) and metabolic (one gene) [38]. Atopy is associated with an increased risk of developing an IgE mediated sensitization to HMW agents [24-26,39,40]. Occupational rhinitis is also associated with OA and rhinitis symptoms precede those of the asthma in 58% of the cases where HMW agents are implicated and in 25% of cases involving LMW [41].

In addition, the large increase in the prevalence of asthma observed in most developed countries [42] during the last decades has pointed toward environmental factors being responsible for part of this increase--particularly changes in diet, such as decreased consumption of fresh fruits and vegetables and unprocessed food [43-45]. Because of the components of airway constriction and inflammation involved in asthma, several nutritional factors that may act on muscle constriction or inflammatory response have been hypothesized to play a role on this disease. Romieu, in a literature review, suggests opposite associations between antioxidant vitamins intake (particularly vitamin C and, to a lesser extent, vitamin E), omega-3 fatty acids or consumption of fresh fruit, and obstructive lung disease [46]. Our attention will also focus on the metabolism of folates, an area (which could play a role in asthma) that remains poorly explored to date. Most studies concerning methyl donors show their involvement in many inflammatory disorders (cardiovascular, neurodegenerative, autoimmune diseases...) [47,48], and some studies have suggested their involvement in the development of atopy. For them, a dietary deficiency of methionine, folate and other B vitamins are associated with a high risk of atopy [49].

## Methods/Design

The ABCD (french acronym for early asthma in bakery and hairdressing sectors) study aims to assess early incidence of OA among young workers according to their sector of activity and exposure duration, and to identify risk factors of OA.

### Study population

This study is a retrospective follow-up with a nested case-control facet. Regarding the cohort study, the exposed population is composed of all bakers, pastry-makers and hairdressers who graduated between 2001 and 2006 from nine vocational schools in Lorraine, North-Eastern France, while the "non-exposed" group comprises all subjects who graduated in 2001 in the sale or the food sectors (butcher, pork butcher, caterer, cook job,...) from the same vocational schools. All those complying with these criteria are contacted by phone to complete a short screening questionnaire to ascertain OA symptoms. All subjects defined as "possibly with OA", based on the answers to this questionnaire (see further), and a matched sample of "non cases" (frequency matching criteria: year of graduation, vocational school and occupational sector) are proposed to be visited to perform a medical examination, spirometry and other tests which are detailed further. The research program is authorized by the Nancy University Hospital ethics committee and written consents are obtained from the young workers themselves.

### Sample size

The calculated sample size aims at answering the two following objectives: (1) to show evidence of an increased incidence during the first years of occupational exercise among young bakers, pastry-makers and hairdressers as compared with young workers without specific occupational exposure; this objective will be pursued with the retrospective cohort study, and (2) to identify some risk factors, including genetic polymorphisms, of this increased incidence, through the nested case-control study. The estimated sample size for the retrospective cohort study should have the power to show a difference in OA incidence between young workers (bakers, pastry-makers and hairdressers) with high seniority (about seven years) and young workers with a low seniority (two years or less). A difference in cumulative incidence of 6% between these two groups (from 1 to 7%) should be demonstrated with a sample of 150 subjects by year, with a power of 80% and an α-risk of 5% (two-tailed test). Further, a sample of 250 young workers with no specific occupational exposure should be used to demonstrate a significant difference of OA incidence after about seven years of activity between bakers, pastry-makers and hairdressers and this "non-exposed" group (expected incidence 3%, α = 5% and 1-β = 75%). The total sample to be investigated in the telephone interview is therefore 1150 young workers. It is expected to provide about 40 possible cases of OA (see further the operational definition of OA in this study). For this number, the nested case-control study would need about 200 controls in order to have the power (1-β = 80%) to detect relevant single nucleotide polymorphisms (SNP) associated with differences in allele frequencies between cases and controls of about 16 to 24% across a set of reasonable allele frequencies among controls (from 5 to 35%; two-tailed test, α = 5%, 1-β = 80%). Similar comparisons can be made for other risk factors with differences in prevalence of this order of magnitude.

### Recruitment procedure

Nine vocational training centres in Lorraine participate in the study. They identified from their archives all apprentices who graduated in bakery, pastry, hairstyle, sale or food sectors between 2001 and 2006, along with the apprentices' or parents' addresses and phone numbers during their last training year. Because of the mobility of the young workers during the first years of their occupational activity (departure from family home in particular), a preliminary step was organized. A postal mail was sent to all parents, comprising an explanatory letter, an information sheet on OA and a reply coupon to be completed and returned with the current address and phone number(s) of the young worker. Up to three reminders (two by mail then one by phone) were sent to maximize the response rate.

A medical questionnaire is also administrated by phone to each worker for whom we have a phone number. Participants are subjects who met the following inclusion criteria: (i) no history of asthma before apprenticeship, and, (ii) for "non-exposed" subjects, not to have worked, before or after apprenticeship, in an occupation at risk of OA.

### OA diagnosis and OA timing

**Figure 1:**
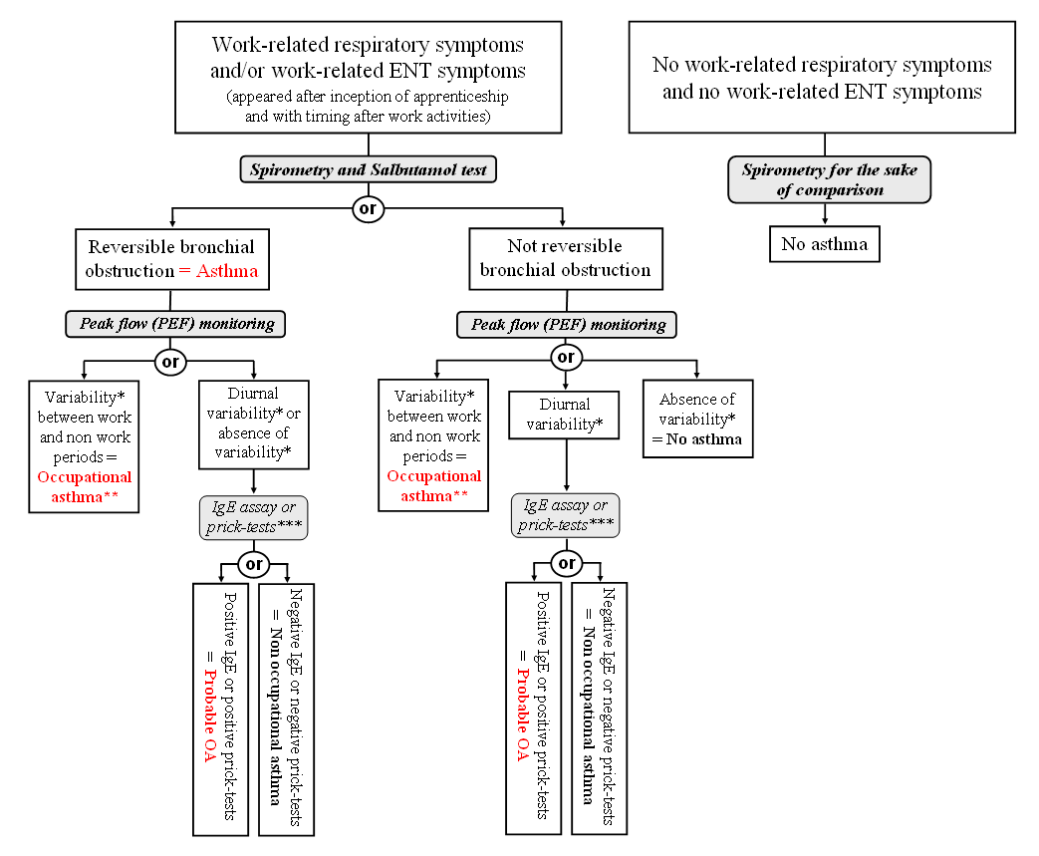
**Decision tree for the definition of occupational asthma**. * Variability is defined by the Oasys-2 score as deterioration during a work period or improvement during a period away from work of at least 20%. ** When only ENT symptoms are present, the diagnosis of infraclinic OA is more appropriate. *** IgE Assay (bakers and pastry makers) and prick-tests (hairdressers) were realized for specific occupational allergens.

The OA diagnosis is made in two times: (i) diagnosing asthma, and (ii) highlighting a temporal relation of symptoms with work activities. The decision tree for defining OA is presented in figure 1. The tests that are undertaken during the medical visit at home to confirm a case of OA will be presented further.

For subjects who present work-related respiratory symptoms and for whom a diagnosis of OA or probable OA is evoked or confirmed by a doctor, the date retained for this diagnosis corresponds to the first occurrence of the respiratory symptoms. On the other hand, for subjects considered as asthmatics (infra-clinical form) because of their results to the tests performed during the visit and their occupational exposure and who present only work-related ENT symptoms, the corresponding date is that of the medical visit.

### Initial telephone screening questionnaire

During the first step of the study, nurses trained for this study administer a short standardized questionnaire by phone concerning exposure duration, respiratory, ENT, skin symptoms and smoking status. This phone interview aims to screen subjects who are likely to exhibit OA and allows classifying them into three categories: "work-related respiratory (+/- ENT) symptoms", "only work-related ENT symptoms" and "absence of work-related symptoms".

Work-related respiratory symptoms are considered present if three conditions are combined: (a) presence of a diagnosed asthma or at least one of the respiratory symptoms (wheezing, breathlessness, chest tightness, cough and sputum) during the last 12 months. [If the person has left his/her activity or was found to take asthma medication, the 12 months period was defined before quitting and/or starting medication intake] (b) the symptoms should have appeared after inception of apprenticeship; and (c) symptoms are present during the working days and improve or disappear during week-ends or holidays.

Work-related ENT symptoms are considered present if three conditions are combined: (a) presence of at least one major symptom of rhinitis (nasal obstruction, runny nose) during the last 12 months (or during the 12 months prior to quitting the activity if the subject has switched to another occupation than the one for which he/she had been trained); (b) the symptoms should have appeared after inception of apprenticeship or, for atopic subjects, ENT symptoms should have been aggravated at work; and (c) symptoms are present during the working days. [Amendment or disappearance of these symptoms during week-ends or holidays is not considered, not to overlook chronic rhinitis with an occupational origin.]

We define all subjects that pertain to one of these two first categories as "possibly OA case". All others are defined as "non cases".

Information concerning exposure (duration of apprenticeship, diplomas obtained, duration of occupational exposure) and smoking status are also collected. Current smokers are defined as subjects who reported smoking on average at least one cigarette a day for at least one year and past smokers are defined as subjects who reported past smoking but who do not smoke any more.

If participation to this interview is declined, we try to retrieve basic data during the phone exchange: information about study inclusion criteria, reasons if he/she has left his/her training sector if relevant, and whether the subject had asthma symptoms or treatment.

### Medical visits

All subjects considered as "possibly OA case" are offered a visit. Also, a random sample of two hundreds "non cases" are selected and offered a medical visit that met two criteria: (i) to be representative of all the subjects who graduated between 2001 and 2006 as to the training sector (bakery and pastry, hairstyle or others), the year of graduation (2001-2002; 2003-2004 or 2005-2006) and the vocational school (graduated in the *Moselle *department - with 4 schools - or others) and (ii) to be representative of all those who complete the phone questionnaire concerning the prevalence of symptoms (work-related respiratory symptoms, work-related ENT symptoms and others).

Prior to the visit (about fifteen days before), a letter is sent to each volunteer, including an explanatory letter and an information sheet on OA, consent forms (to be completed and returned) and a food consumption questionnaire (to be completed and brought back during the visit).

Two persons, including a physician, participate to each visit (TR and DSA or TR and an intern). The duration of each visit is approximately one hour and a half. The examinations take place in this following order:

#### 1- Clinical examination and questionnaire

The clinical history is evaluated with a standardized questionnaire with a view to check and complement the data collected during the phone screening questionnaire: personal and family antecedents, exploration of symptoms associated with a variety of exposures (environmental tobacco smoke, air pollution, dust, ...), past and present smoking habits, details on respiratory, ENT and skin symptoms. Medical antecedents are looked for (respiratory and ENT diseases, allergic conditions), and pathologies which contraindicate the bronchodilator reversibility test (hyperthyroidism, cardiac conditions). More detailed data on smoking are collected: number of cigarettes smoked, duration of smoking.

A clinical examination, including pulmonary auscultation centred on pulmonary, ENT, ophthalmologic and skin symptoms is then conducted, searching for sibilants, signs of conjunctival irritation, nasal or bronchial obstruction or atopic diseases. Weight, size and blood pressure are recorded.

#### ...and verification of the food consumption questionnaire

Due to the importance of dietary factors in aetiopathogenic hypotheses of asthma, diet is assessed by a food frequency questionnaire (SUVIMAX 2: http://clinicaltrials.gov/ct2/show/NCT01096537). It is sent by mail to all subjects who accepted the medical examination and is collected during the visit and verified. It will in particular be used to qualitatively evaluate dietary imbalance of nutritional factors of interest: vitamin B (folates, B12, B6, B2), polyphenols, carotenoids, vitamins E and C, omega-3 polyunsaturated fatty acids (PUFA).

#### 2- Occupational exposure questionnaire

To quantify occupational exposure, a questionnaire is completed during the visit. Three distinct questionnaires are used: one for bakers and pastry-makers, one for hairdressers and the other for the referent population. The two former questionnaires have been devised after an exposure study among apprentices in their workplace [50,51] and comprise different sections: occupational history, work tasks, occupational environment and safety devices; the last section allows identifying allergens that may be encountered in the workplace. For "non-exposed" workers, the questionnaire only comprises the first and the last section.

#### 3- Exhaled NO and CO measurements

To complete the smoking status, CO is measured by a portable analyzer (Micro CO^® ^analyser, Micro Medical, United Kingdom) that provides values after a single expiration.

Because FE_NO _has been shown by our team as an early marker of airways inflammation among bakery and hairdressing apprentice [52], the Fractional Exhaled Nitric Oxide (FE_NO_) is measured by a portable analyzer (NioxMino^® ^analyzer, Aerocrine, Sweden). All participants will undergo the measurement of FE_NO _using the NIOX analyzer at a mouth flow rate of 50 mL/s and a pressure of 10 cm H_2_O. A single measurement is then undertaken immediately afterward using the portable MINO device with identical mouth flow rate and pressure settings.

#### 4- Spirometry

After CO and FE_NO _measurements, spirometry is performed in the standing position using Spirolab II (RDSM Co., Italy). Forced vital capacity (FVC), forced expiratory volume in one second (FEV1) and peak expiratory flow rate at various percentages of vital capacity (V'max) are obtained during forced expiration from total lung capacity. At least three forced expirations satisfying the American Thoracic Society (ATS) criteria [53] are performed. The highest values for FVC and FEV1 are used for analysis. Results are expressed as percentage of predicted values using the equations recommended by the European Respiratory Society (ERS) [54]. The ATS/ERS norms are used for results interpretation [55]. For all subjects with work-related symptoms, a reversibility test is realised (inhalation of four separate doses of 100 mg of salbutamol). Known hypersensitivity to one of the ingredients of Salbutamol and a contraindication to the use of this drug (heart failure, hypertension, diabetes, etc.) don't allow the realization of this test. Reversibility is defined by an increase of FEV_1 _by at least 12% and at least 200 ml compared with baseline during a single test session [55,56].

#### 5- Blood sampling

Blood samples are taken to determine total and specific IgE by the Phadia method (wheat flour, α-amylase and baker's yeast, quaternary ammoniums, persulfates, and also common allergens to evaluate the atopic status); circulating concentrations of vitamin B (folate, B12), carotenoids, vitamin E, omega 3 fatty acids; inflammation and lipid parameters [57] (triglycerides, phospholipids, total cholesterol, HDL-cholesterol, C reactive protein, TNFα and IL1β); metabolic markers (homocysteine) [58]; and markers of oxidative stress (erythrocyte SOD1, glutathione peroxidase, glutathione reductase, 4-hydroxynonenal, malondialdehyde). Two 5 to 7 ml EDTA tubes and one 5 ml tube of serum are collected for this purpose.

In addition, due to their potential role in our pathophysiological model, a number of genetic variants have been preselected from a search on the SNP NCBI database and Hapmap: (i) variants with a phenotypic expression and/or associated with functional alterations related to asthma and (ii) variants related to asthma and with an allele frequency > 10%. These determinants are studied on the genotyping platform Illumina^® ^BeadXpress of Inserm U954, which allows to genotype 300 SNPs on a macro-array. In addition, we also look for polymorphisms that have already been studied in the asthma and bronchial inflammation literature: TNFα [59], IL-1 cluster (IL1α, IL1β, and IL1RN) [60,61]. Regarding bioavailability and metabolism of n-3 fatty acids and polyphenols, we study the polymorphisms of gene involved in blood and cellular transport, including Apo AIV, APO E, CETP, HL-480, I- and L-FABP, LPL, MTP, PLTP, ABC, SR-B1, ABCA1 and 3, and CD36. And finally, concerning metabolism of homocysteine, we study a selection of 380 gene variants involved in the cellular metabolism of folate and cycle of methionine and in the absorption and transport of folate and vitamin B12, including variants from MTHFR, MTR, MTRR, TCN2, GIF, RFC and COMTs.

#### 6- Peak-Flow monitoring

All subjects who report work-related respiratory and/or ENT symptoms are asked to monitor peak expiratory flow rate (PEFR) for three weeks [62]. They are given a peak-flow meter (Asma-1^®^, Vitalograph, Ennis, Ireland), a results sheet, and instructions on how to use the device. They have to measure their PEFR four times a day: two on the workplace (the first one 2-3 hours after the beginning and the second one at the end of the work shift), and two out of work (one before going to work and the other at bedtime) [63,64]. For each measurement, the best value of three attempts is kept. PEFR variability is calculated between values at work and out of work, for each day, during the three weeks of the follow-up. An increase of PEFR variability is considered to be clinically relevant when the relative frequency of days with a 20% variation of PEFR differs by at least 10% between days at work and days off work. The computer program *Oasys*-*2 *is used to plot and interpret serial PEFR readings: curves connecting the points are used to construct curves of the time-course of PEFR. The Oasys work effect index gives a score between 1 and 4 (or zero if nothing to score). Scores above 2.5 have 94% specificity for occupational asthma and 75% sensitivity when using independent methods of diagnosis, so a negative score cannot be used to exclude occupational asthma [65].

#### 7- Skin prick-tests (optional)

The hairdressers, whom diagnosis of OA is evoked but not confirmed by the peak-flow monitoring, will be directed to a specialized structure to perform skin prick-tests with occupational allergens.

### Data capture and analysis

Data are entered in a database by optical scanning device. Additional visual control of these entries is realised by a technician. The analysis comprises several steps. After description of the whole study population and of subjects who had the medical visits, the overall OA incidence will be compared across the job sectors. Among bakers and pastry makers or hairdressers, OA incidence will be computed according to seniority. Finally, a nested case-control logistic regression analysis will be used to identify risk factors of OA (nutritional, genetic polymorphisms or others factors such as tobacco smoking).

## Discussion

### Study design and study population

This study includes six groups of subjects of increasing age and duration of exposure since termination of apprenticeship across different jobs. Exposure duration has been evoked as a determinant of asthma in occupations at risk [23,66]. This study strives to quantify this association according to exposure duration to allergens and irritants. Some subjects might quit soon, because of airways impairment and allergies, a phenomenon that underlines the relevance of studying the early times since inception of exposure [67,68].

Not to require an authorization from employers, medical visits take place at home, before or after the work shift, during week-ends or holidays. This setting, where subjects are not seen in their exposure situation, might reduce the capacity of the study to identify bronchial obstruction induced by exposure to occupational allergens and/or irritants. This problem is also encountered for the interpretation of FE_NO _measurement, which might underestimate the true prevalence of bronchial inflammation. For this reason, peak expiratory flow rate (PEFR) surveillance is realized for all participants presenting evocative symptoms, irrespective of other tests.

This study includes successive cohorts of increasing time since engaging in the occupations of interest. In each cohort the data that are collected combine cross-sectional information (clinical and biological data collected during the medical visit), and historical information (reconstruction of the clinical history and of the exposure at work in a retrospective manner). This methodology might suffer from a memorization bias. However, the young age of our population is likely to reduce this limitation. We define the date of OA diagnosis as the first occurrence of respiratory symptoms at work; it is the date of the visit, when dealing with subjects with no respiratory symptom. This is done not to overestimate the latency period, i.e. the time between the start of occupational exposure and occurrence of OA, a bias that affects a majority of the studies which date asthma onset according to when it was diagnosed.

Subjects who, after completion of their apprenticeship, found a job out of the Lorraine region could not be seen, because the distance precluded visiting them. There is no reason why this subgroup (which represents approximately 10 per cent of subjects considered as "at risk") should exhibit an asthma incidence pattern different from that of their counterparts who set in the region. To avoid underestimation of OA incidence, the number of OA cases will be assessed in this subgroup using the data from visits.

For the "non-exposed" job categories, we have selected young workers who graduated in the same vocational schools in order to have similar socio-demographic profiles, thus reducing the influence of factors other than occupational. The selected jobs are from the sales or the food sectors, which represent a large number of occupations after apprenticeship in France.

### Relevance of the project

In addition to the care given to the reconstruction of the occupational history of each subject since inception of apprenticeship, this study tackles a large number of determinants of OA in "at risk" occupations. The greatest prevalences are observed in France among bakers and pastry makers, but increasing number of cases was seen among women over the last years, in particular in hairdressers [3]. Bakery and, to a lesser degree, pastry making, entail exposure to high concentrations of flour dust and to fungal α-amylase enzyme, which have sensitising properties. Bakers and pastry makers also experience peak and short-lasting exposures (minutes) [69,70] (Mounier-Geyssant E, Massin N, Paris C, Zmirou-Navier D: Longitudinal analysis of activities inducing peak exposures to flour dust among bakery and pastry apprentices, submitted). Albin et al. [21] showed a slightly, but not significantly, higher incidence of asthma in hairdressers that often perform hair bleaching treatments or use hair sprays compared with more infrequent users, and Akpinar-Elci et al. [71] found a higher risk of occupational asthma in high work intensity hairdressers. On the other hand, Iwatsubo et al were unable to describe a specific hairdressing activities related to the deterioration of lung function they found among hairdressing apprentices during a 3-year follow-up [72].

A relationship between diet and asthma has also been described. The "nutritional hypothesis" ascribes the increase in respiratory allergies to changes in dietary intake, principally of anti-oxidants and lipids [73,74]. Anti-oxidants and omega 3 PUFA may have beneficial effects on the incidence of asthma and on atopic diseases [73]. Since asthma in adults is associated with a low intake of fruits, nutritional anti-oxidants, vitamin C, and magnesium, it has been suggested that the diet is a potentially modifiable risk factor for the development of asthma [75]. Several epidemiological studies demonstrated that dietary vitamin C intake or serum ascorbate is positively associated with ventilatory function in children and adults [76,77]. Positive effects have also been shown for dietary vitamin E on lung function [76-79] and possibly on asthma and atopy [80,81]. However, the effectiveness of asthma and respiratory allergies prevention through dietary supplementation is still debated [82].

This study will allow investigating a variety of gene known to be involved in disease and gene-gene interactions. No study has previously, to our knowledge, considered simultaneously the nutrigenetic, nutrigenomic and metabolic interactions regarding the association between genetic determinants and airways inflammation.

The interaction between occupational exposure and nutrition modulated by certain SNP of genes involved in the production of proinflammatory factors or in the metabolism of B group vitamins has not yet been studied and would therefore be an original aspect of this project. This might help elucidate, if any, differential incidences of early asthma in these high-risk occupations. Hence, in phase with a recent editorial call in the European respiratory Journal [83], this project is highly multidisciplinary, associating an occupational health perspective -as occupational asthma constitutes a serious health problem - and a mechanistic perspective, with exploration of the metabolic processes involved in oxidative stress and immunological sensitization induced by occupational exposure, and of some of their genetic determinants.

### Asthma diagnosis

The diagnosis of asthma is usually based on the presence of characteristic symptoms such as episodic breathlessness, wheezing, cough and chest tightness [84]. Now, the medical examination serves to exclude rather than to confirm the diagnosis of OA [85]. Measurement of lung function is also recommended. Because our study takes place in the field, bronchial challenge to metacholine, a test which is considered as the gold standard, is not practicable. Assessing whether an obstructive syndrome is reversible after bronchodilatator inhalation (here, Salbutamol) is an important step in the diagnosis of asthma. Hence, our definition of asthma is based on the presence of a reversible bronchial obstruction as described in the Global Initiative for Asthma [54]. Now, many asthmatic subjects may have normal or near-normal pulmonary function, especially at distance from exposure and during non exacerbation periods [86]. To account for this, we chose to perform peak-flow monitoring for all subjects who report work-related respiratory or ENT symptoms. A daily PEFR amplitude (measured as the highest daily value minus the lowest daily value) greater than 20% supports the diagnosis of asthma [54,87]. Several researchers have suggested that serial measurements of PEFR at the workplace and away from the workplace is an objective measure and an appropriate first step in establishing a causal relationship between work and lung function alteration, or asthma [62,65,88]. The Oasys quality critera for a serial peak flow record is defined as being of adequate quality if it lasts at least 2.5 weeks, with at least 4 readings per day and 3 consecutive workdays in each work period. In this setting, the sensitivity and specificity of adequate records were respectively 78.1% and 91.8%, versus 63.6% and 83.3% for inadequate records [62]. While serial PEFRs have a number of advantages as a tool for diagnosing sensitizer-induced OA, they also have some limitations. They are effort dependent and require good cooperation from subjects being investigated. PEFRs may be incomplete or uninterpretable for a variety of reasons [89,90]. In case of doubt concerning the occupational character of the asthma, other examinations (IgE assays or Prick-tests) will be used for the diagnosis.

Some subjects had stopped to work in the sector they had been trained for. Excluding them might yield underestimation of the frequency of OA if some quit because they experienced untoward consequences of the exposures of interest. So, they remain in our study but only the symptoms during the 12 months prior to quitting are considered. However, for these subjects, serial measurements of PEFR cannot any more bring to light variability between measurements at the workplace in their initial job and away from the workplace. Only diurnal variability can be recorded. For such subjects who would present work-related respiratory symptoms (as defined in our study) associated with diurnal PEFR variability, dosage of specific IgE is used to assess the association between occupational exposure and symptoms. Positive cases are considered as "probable OA". This test is less relevant for hairdressers, in absence of a demonstrated underlying IgE mediated mechanism for persulphates [91]. Unfortunately, the University hospital requires that skin prick-tests be done in a medical environment, thus precluding their realization in the study setting. Because of these different limitations, hairdressers can be invited to ask their general physician to realize skin prick tests (commons allergens, persulfates, latex, and sericine) and to report their results.

## Conclusions

This study may allow describing a latent period between inception of exposure and the rise of the prevalence of asthma symptoms and to compare this time pattern according to the nature of exposure, an information that would be useful for the prevention of OA. Such a period would be suited to undertake screening campaigns of this emerging asthma at a stage when occupational hygiene measures and adapted therapeutic interventions, might be effective.

## Abbreviations

OA: occupational asthma; ENT: Ear-Nose-Throat; FE_NO_: fractional exhaled nitric oxide; CO: carbon oxide; IgE: immunoglobulin E; RADS: reactive airways dysfunction syndrome; HMW: high molecular weight; LMW: low molecular weight; IRR: incidence ratio rate; ONAP: observatoire national des asthmes professionnels; ABCD: asthme en boulangerie et coiffure débutant; SNP: single nucleotide polymorphism; PUFA: polyunsaturated fatty acids; FVC: forced vital capacity; FEV_1_: forced expiratory volume in 1 second; V'max: maximal expiratory flows at various lung volumes; ATS: american thoracic society; ERS: european respiratory society; PEFR: peak expiratory flow rate.

## Competing interests

The authors declare that they have no competing interests.

## Authors' contributions

TR carried out the epidemiologic study, drafted the manuscript and participates in the medical visits with VC and DSA for data collection; he will perform the statistical analysis. TR, DS, JLG, RMG participated in the design of the protocol. CP and DZN designed the study and drafted the manuscript. All authors read and approved the final manuscript.

## Pre-publication history

The pre-publication history for this paper can be accessed here:

http://www.biomedcentral.com/1471-2458/10/206/prepub
